# ECG Patient Simulator Based on Mathematical Models

**DOI:** 10.3390/s22155714

**Published:** 2022-07-30

**Authors:** Mario Alan Quiroz-Juárez, Juan Alberto Rosales-Juárez, Omar Jiménez-Ramírez, Rubén Vázquez-Medina, José Luis Aragón

**Affiliations:** 1 Centro de Física Aplicada y Tecnología Avanzada, Universidad Nacional Autónoma de México, Boulevard Juriquilla 3001, Queretaro 76230, Mexico; jlaragon@unam.mx; 2 Instituto Politécnico Nacional, Escuela Superior de Ingeniería Mecánica y Eléctrica, Santa Ana 1000, San Francisco Culhuacán, Mexico City 04430, Mexico; jrosalesj1600@alumno.ipn.mx (J.A.R.-J.); ojimenezr@ipn.mx (O.J.-R.); 3 Instituto Politécnico Nacional, Centro de Investigación en Ciencia Aplicada y Tecnología Avanzada, Cerro Blanco 141, Colinas del Cimatario, Queretaro 76090, Mexico; ruvazquez@ipn.mx

**Keywords:** ECG simulator, biomedical engineering, cardiac dynamics, synthetic ECG generation, embedded system

## Abstract

In this work, we propose a versatile, low-cost, and tunable electronic device to generate realistic electrocardiogram (ECG) waveforms, capable of simulating ECG of patients within a wide range of possibilities. A visual analysis of the clinical ECG register provides the cardiologist with vital physiological information to determine the patient’s heart condition. Because of its clinical significance, there is a strong interest in algorithms and medical ECG measuring devices that acquire, preserve, and process ECG recordings with high fidelity. Bearing this in mind, the proposed electronic device is based on four different mathematical models describing macroscopic heartbeat dynamics with ordinary differential equations. Firstly, we produce full 12-lead ECG profiles by implementing a model comprising a network of heterogeneous oscillators. Then, we implement a discretized reaction–diffusion model in our electronic device to reproduce ECG waveforms from various rhythm disorders. Finally, in order to show the versatility and capabilities of our system, we include two additional models, a ring of three coupled oscillators and a model based on a quasiperiodic motion, which can reproduce a wide range of pathological conditions. With this, the proposed device can reproduce around thirty-two cardiac rhythms with the possibility of exploring different parameter values to simulate new arrhythmias with the same hardware. Our system, which is a hybrid analog–digital circuit, generates realistic ECG signals through digital-to-analog converters whose amplitudes and waveforms are controlled through an interactive and friendly graphic interface. Our ECG patient simulator arises as a promising platform for assessing the performance of electrocardiograph equipment and ECG signal processing software in clinical trials. Additionally the produced 12-lead profiles can be tested in patient monitoring systems.

## 1. Introduction

In the cardiac cycle, the so-called systolic and diastolic phases refer to the state of contraction and relaxation in the heart, respectively. Electrical impulses arising from transmembrane ionic currents governs these phases [1,2,3]. As a consequence of this bioelectrical activity, variations in the skin’s electrical potential are produced, which can be measured with highly sensitive instruments known as electrocardiographs and electrodes placed at predefined points on the skin surface [4,5,6]. The recording of these potential differences is known as an electrocardiogram (ECG) and represents a clinical tool widely used by cardiologists in routine medical evaluations to determine the pathophysiological conditions of the patient [7,8,9,10,11].

Because of the great significance of ECG waveforms in the diagnosis of cardiovascular diseases, there has been a significant amount of research effort for ensuring that medical electrocardiogram measuring devices [12,13] and biomedical signal processing algorithms [14,15,16] acquire, preserve, and process ECG recordings with high fidelity. In this context, the synthetic ECG patient simulators have emerged as useful tools to test, evaluate, and calibrate electrocardiograph equipment and ECG signal processing software [17,18,19,20,21,22,23,24,25]. In fact, ECG simulators have been proposed not only as a worthwhile tool for equipment and algorithm testing but also as a powerful instrument to assist in the teaching of ECG diagnosis. With its help, ECG interpretation skills of undergraduate medical students can be improved, mainly because a low performances of this medical skill has been reported between students, residents, and physicians [26,27]. Of course, ECG misinterpretations can expose the patient to improper prescription and delays in the right treatment [28,29].

Hitherto, unfortunately, ECG simulators do not fulfill all desirable features of a universal simulator because they show shortcomings in amplitudes, waveforms, and time-intervals of the produced signals. Additionally, most ECG simulators need an internal database of sampled healthy and pathological ECG waveforms. The main disadvantage of these implementations is that the variety of ECG waveforms they produce is limited by the device’s storing capability, so they are not a useful tool for a dynamic interpretation of abnormalities in the heart.

In order to overcome these shortcomings, and in light of the importance of mathematical modeling on the understanding of complex biological processes involved in the cardiac conduction system [30,31,32,33,34,35,36], in this work, we propose an ECG patient simulator that implements four different mathematical models to generate realistic ECG waveforms within a broad range of possibilities. Our ECG patient simulator is an electronic device that comprises a hybrid analog–digital circuit formed by 32-bit microcontrollers, digital-to-analog converters (DACs), and general-purpose operational amplifiers (OPAMPs), with the possibility of controlling the amplitudes, waveforms, and parameters of the mathematical models through an interactive and friendly graphic interface. Additionally, the used mathematical models in our electronic device have the advantage of allowing us to explore different parameter conditions to simulate new arrhythmias with the same hardware.

To mimic clinical 12-lead ECG waveforms, we implement an extended heterogeneous oscillator model of the cardiac conduction system [37], whose heart rate can be driven via software. We also implement a discretized reaction–diffusion model [38], a ring of three coupled oscillators [39], and a model based on quasiperiodic motions [40] for reproducing ECG with a great variety of rhythm disorders. Given this, we consider that our electronic device is a useful tool for research, medical education, and clinical testing purposes. In particular, the proposed simulator can be used to assess the performance of electrocardiograph equipment and ECG signal processing software in clinical trials.

## 2. Materials and Methods

As already mentioned, most ECG patient simulators use, as a core generator, an internal database comprising sampled healthy and pathological ECG waveforms, which are then analogically played back by a digital-to-analog converter. To avoid this, we implement four different models describing macroscopic heartbeat dynamics with a set of ordinary differential equations, all of them capable of reproducing synthetic ECG waveforms under normal and pathological conditions. In what follows, we briefly review each of the models implemented in the proposed ECG patient simulator.

### 2.1. The Models

#### 2.1.1. Heterogeneous Nonlinear Oscillators

A general and useful model to reproduce full 12-lead ECG waveforms is the extended heterogeneous oscillator model of the cardiac conduction system [37]. The first version of this model was proposed by [41], and later, it was applied to simulate the appearance of ventricular fibrillation as an instance of chaos [42]. The model consists of main natural pacemakers represented by modified Van der Pol oscillators [43,44,45], and electrical response of atrial and ventricular muscles (depolarization and repolarization processes) modeled by modified FitzHugh–Nagumo equations [46,47,48].

Natural pacemakers, namely, sinoatrial (SA) node, atrioventricular (AV) node, and His–Purkinje (HP) system, are given by the following sets of ordinary differential equations:

(1) $$\begin{array}{c} {\dot{x}}_{i}=y_{i},\\ {\dot{y}}_{i}=-a_{i}\left(x_{i}^{2}-u\right)y_{i}-f_{i}x_{i}\left(x_{i}+d\right)\left(x_{i}+e_{i}\right)+K_{node}\left(y_{i-1}^{\tau_{node}}-y_{i}\right), \end{array}$$

where $x_{i}$ stand for action potentials, i=1,2,3 corresponds to SA, AV and HP nodes, respectively. $K_{node}$ denotes the particular coupling constant for each pacemaker, $y_{i}^{\tau_{\mathrm{node}}}=y_{i}\left(t-\tau_{node}\right)$ are the time-delayed coupling components, and $\tau_{\mathrm{node}}$ is the corresponding coupling time delay. The terms $a_{i}$, $f_{i}$, *u*, *d*, and $e_{i}$ are the parameters of each oscillator.

The description of the electrical responses of AT and VN muscles are described by:

(2) $$\begin{array}{c} {\dot{z}}_{j}=k_{j}\left(-c_{j}z_{j}\left(z_{j}-w_{j1}\right)\left(z_{j}-w_{j2}\right)-b_{j}v_{j}-g_{j}v_{j}z_{j}+I_{j}\right),\\ {\dot{v}}_{j}=k_{j}h_{j}\left(z_{j}-v_{j}\right), \end{array}$$

with j=1,…,4, which refers to the P-wave, Ta-wave, QRS complex, and T-wave, respectively.

The magnitude of the stimulation current that couples the SA and HP pacemaker to AT and VN muscles are, respectively, $I_{j}=C_{j}Y_{j}H\left(Y_{j}\right)$, where $C_{j}$ are the corresponding coupling coefficients, H(Y) is the step function, and $Y_{1}=y_{1}$, $Y_{2}=-y_{1}$, $Y_{3}=y_{3}$, and $Y_{4}=-y_{3}$ from (1). The parameters $k_{j}$, $c_{j}$, $w_{j1}$, $w_{j2}$, $b_{j}$, $h_{j}$, and $g_{j}$ control the rest state, the excitability, the duration of the action potential, the excitation threshold, and the excited state of each oscillator.

The net ECG(t) waveform is calculated as a composition of muscle electrical responses in the following way:

(3) $$ECG\left(t\right)=z_{0}+\alpha_{1}z_{1}-\alpha_{2}z_{2}+\alpha_{3}z_{3}+\alpha_{4}z_{4},$$

where $z_{0}$ is the baseline value of ECG(t), and $\alpha_{1}\ldots \alpha_{4}$ are the weighting coefficients for each lead, which are given in [37].

It is worth saying that this model can reproduce several well-known rhythm disorders, such as tachycardia, bradycardia, complete SA–AV block, and AV–HP block, by implementing the parameter values reported in [41].

#### 2.1.2. Reaction–Diffusion Model Spatially Discretized

Recently, a model based on a discretized reaction–diffusion system to reproduce electrocardiograms from healthy hearts and various rhythm disorders was introduced [38]. Since the model presents chaotic behavior, it was associated with ventricular fibrillation, which arises from a normal rhythm through the so-called Ruelle–Takens–Newhouse scenario [49], as experimental studies suggest [50,51].

The model comprises a set of three nonlinear oscillators obtained from the spatial discretization of the BVAM model [52], whose variables simulate the main pacemakers in the heart. The model consists of the following ordinary differential equations:

(4) $$\begin{array}{c} {\dot{x}}_{1}=x_{1}-x_{2}-Cx_{1}x_{2}-x_{1}{x_{2}}^{2},\\ {\dot{x}}_{2}=Hx_{1}-3x_{2}+Cx_{1}x_{2}+x_{1}{x_{2}}^{2}+\beta \left(x_{4}-x_{2}\right),\\ {\dot{x}}_{3}=x_{3}-x_{4}-Cx_{3}x_{4}-x_{3}{x_{4}}^{2},\\ {\dot{x}}_{4}=Hx_{3}-3x_{4}+Cx_{3}x_{4}+x_{3}{x_{4}}^{2}+2\beta \left({x_{2}-x}_{4}\right). \end{array}$$

Here, *H* and *C* are parameters controlling the network’s dynamical behavior, and β is the local interaction between oscillators. ECG waveforms can be generated by a linear mixing of the variables $x_{i}$, as follows:

(5) $$ECG\left(t\right)=K_{1}x_{1}+K_{2}x_{2}+K_{3}x_{3}+K_{4}x_{4}.$$

Sinus rhythm, sinus/ventricular tachycardia, atrial/ventricular flutter, and ventricular fibrillation can be reproduced by varying the parameters *H*, *C*, and $K_{i}$ as described in [38].

#### 2.1.3. Ring of Three-Coupled Oscillators

A relatively old idea was to consider the heart as a system of nonlinear coupled oscillators. One of the pioneering studies was developed by Van der Pol (VdP) and Van der Mark (VdM) [53]. In [39], a model is proposed consisting of three modified VdP oscillators [43,44,45] that represent the main pacemakers in the heart. This model consists of six ordinary differential equations coupled with time delays:

(6) $$\begin{array}{c} \dot{x_{1}}=x_{2},\\ \dot{x_{2}}=-a_{SA}x_{2}\left(x_{1}-w_{{SA}_{1}}\right)\left(x_{1}-w_{{SA}_{2}}\right)+\rho_{SA}\sin\left(\omega_{SA}t\right)\\ \;\;-x_{1}\left(x_{1}-d_{SA}\right)\left(x_{1}-e_{SA}\right)-k_{SA-AV}\left(x_{1}-x_{3}^{\tau_{SA-AV}}\right)\\ \;\;-k_{SA-HP}\left(x_{1}-x_{5}^{\tau_{SA-HP}}\right),\\ \dot{x_{3}}=x_{4},\\ \dot{x_{4}}=-a_{AV}x_{4}\left(x_{3}-w_{{AV}_{1}}\right)\left(x_{3}-w_{{AV}_{2}}\right)+\rho_{AV}\sin\left(\omega_{AV}t\right)\\ \;\;-x_{3}\left(x_{3}-d_{AV}\right)\left(x_{3}-e_{AV}\right)-k_{AV-SA}\left(x_{3}-x_{1}^{\tau_{AV-SA}}\right)\\ \;\;-k_{AV-HP}\left(x_{3}-x_{5}^{\tau_{AV-HP}}\right),\\ \dot{x_{5}}=x_{6},\\ \dot{x_{6}}=-a_{HP}x_{6}\left(x_{5}-w_{{HP}_{1}}\right)\left(x_{5}-w_{{HP}_{2}}\right)+\rho_{HP}\sin\left(\omega_{HP}t\right)\\ \;\;-x_{5}\left(x_{5}-d_{HP}\right)\left(x_{5}-e_{HP}\right)-k_{HP-SA}\left(x_{5}-x_{1}^{\tau_{HP-SA}}\right)\\ \;\;-k_{HP-AV}\left(x_{5}-x_{3}^{\tau_{HP-AV}}\right). \end{array}$$

The terms $a_{node}$, $w_{{node}_{i}}$, $d_{node}$, and $e_{node}$ are the parameters of each oscillator, where the index i=1,2 and node corresponds to SA, AV, and HP nodes. $k_{SA-AV}$, $k_{SA-HP}$, $k_{AV-SA}$, $k_{AV-HP}$, $k_{HP-SA}$, and $k_{HP-AV}$ denote the particular coupling constant for each pacemaker. The transport delay terms are given by $\tau_{SA-AV}$, $\tau_{SA-HP}$, $\tau_{AV-SA}$, $\tau_{AV-HP}$, $\tau_{HP-SA}$, and $\tau_{HP-AV}$. The amplitudes and frequencies of the periodic driving terms are $\rho_{SA}$, $\rho_{AV}$, $\rho_{HP}$ and $\omega_{SA}$, $\omega_{AV}$, $\omega_{HP}$, respectively.

The ECG waveforms are obtained from a linear combination of the variables:

(7) $$ECG\left(t\right)=\left(\alpha_{0}+\alpha_{1}x_{1}+\alpha_{3}x_{3}+\alpha_{5}x_{5}\right)\beta_{G},$$

where $\beta_{G}$ is a scaling factor

The parameter values used as reference for reproducing sinus rhythm, ventricular flutter, bradycardia and ventricular fibrillation are suggested in [39].

#### 2.1.4. Extended Dynamical Model Based on a Quasi-Periodic Motion

Sayadi et al. [40] developed a Gaussian wave-based state-space to reproduce the temporal dynamics of the ECG waveform, based on a modification of the model proposed in [54]. They showed that their model may be effectively used for generating synthetic ECG waveforms, as well as characteristic waves, such as the atrial and ventricular complexes, i.e., P, QRS, and T.

Assuming the presence of three distinct characteristic waves corresponding to the *P* wave, QRS complex, and *T* wave, the ECG waveform is divided into three coupled components, each related to a specific portion of the heart cycle. The proposed mathematical model is:

(8) $$\begin{array}{c} \dot{x}=\alpha x-\omega y,\\ \dot{y}=\alpha y-\omega x,\\ \dot{P}=-\sum_{i\in \left\{P^{-},P^{+}\right\}}^{}a_{i}\Delta \theta_{i}e^{\left(-\frac{\Delta \theta_{i}^{2}}{2b_{i}^{2}}\right)}-\left(P-P_{0}\right),\\ \dot{C}=-\sum_{i\in \left\{Q,R,S\right\}}^{}a_{i}\Delta \theta_{i}e^{\left(-\frac{\Delta \theta_{i}^{2}}{2b_{i}^{2}}\right)}-\left(C-C_{0}\right),\\ \dot{T}=-\sum_{i\in \left\{T^{-},T^{+}\right\}}^{}a_{i}\Delta \theta_{i}e^{\left(-\frac{\Delta \theta_{i}^{2}}{2b_{i}^{2}}\right)}-\left(T-T_{0}\right), \end{array}$$

where $a_{i}$, $b_{i}$ and $\theta_{i}$ are the parameters of the Gaussian kernels for each characteristic waveform. *P*, *C*, and *T* represent the P-wave, the QRS complex, and the T-wave, respectively, and the +/− superscripts in *P* and *T* denote the two Gaussian waves used to handle asymmetries. $P_{0}$, $C_{0}$, and $T_{0}$ are the baseline values, which are assumed to be coupled to the respiratory frequency $f_{r}$, using:

(9) $$P_{0}\left(t\right)=C_{0}\left(t\right)=T_{0}\left(t\right)=A\sin\left(2\pi f_{r}t\right),$$

The model has seven events ($P^{-}$, $P^{+}$, *Q*, *R*, *S*, $T^{-}$, $T^{+}$), that act as push–pulls in the *z*-direction as the corresponding trajectory passes around the unit limit cycle in the (x,y) plane. To simulate the quasi-periodicity of the cardiac cycle, the time dependent angular frequency of motion around the limit cycle is obtained by applying the same spectral estimation strategy as in [54], where, in that reference, $a_{i}$ were replaced by $a_{i}=\frac{\alpha_{i}}{b_{i}^{2}}$. Finally, the synthetic ECG is obtained as follows:

(10) ECG(t)=P(t)+C(t)+T(t).

Since the model (8) has a large number of free parameters, it is possible to control the morphological features of the synthetic ECG as described in [40]. This feature allows abnormal morphological changes and several pathological conditions.

In what follows, the main components of the proposed ECG patient simulator are described, that is, the electronic circuits (hardware) and the main algorithms used to solve the mathematical models and display results (software).

### 2.2. Hardware

**Figure 1:**
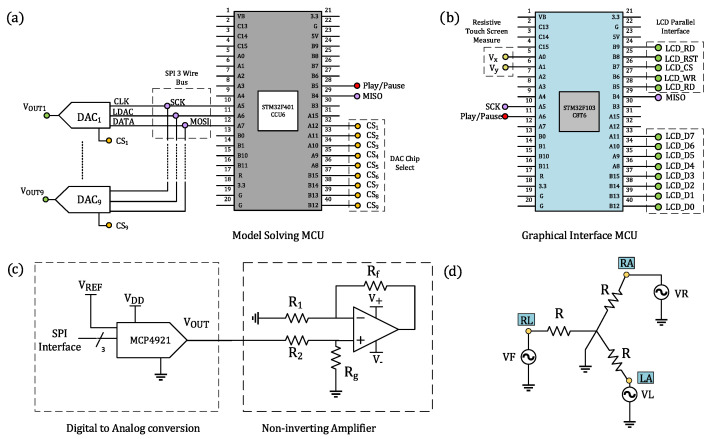
Electronic circuit of the ECG patient simulator. Schematics for (**a**) the STM32F401CCU6 microcontroller, managing the generation of ECG waveforms; (**b**) the STM32F103C8T6 microcontroller, driving the communication protocol with the TFT LCD touch screen; (**c**) digital-to-analog conversion and amplitude regulation of synthetic ECG waveforms; and (**d**) Wye resistor network used to generate electrical potentials from limb electrodes.

The proposed electronic circuit of the ECG patient simulator is shown in Figure 1. The hardware comprises two 32-bit microcontrollers (MCU), manufactured by ST Electronics with series STM32F401CCU6 and STM32F103C8T6; digital-to-analog converters (DAC) MCP4921; general-purpose operational amplifiers (OPAMP) LF353; and a Wye resistor network.

The STM32F401CCU6 is a low-cost, high-performance microcontroller of the family ARM Cortex-M4 Cores, which operates at 84 MHz and includes standard communication peripherals, such as SPI, I2C, USB, USART, and CAN. It also incorporates a single-precision floating-point unit, useful for performing calculations in short timing, a set of digital signal processing instructions, and two analog-to-digital converters. This microcontroller is the central core of the proposed ECG patient simulator, responsible for numerically solving, in real-time, the mathematical models and for interfacing via SPI protocol with nine 12-bit DACs, as shown in Figure 1a. These DACS are used to produce a full 12-lead ECG profile, i.e., precordial leads and bipolar and augmented limb leads. We configure the DACs to operate with an external voltage reference and a clock frequency up to 20 MHz provided by the MCU. A stabilized DC power supply provides the required external voltage reference (+5 V) to the DACs.

Our ECG patient simulator implements a 320 × 240-pixel color TFT LCD touch screen, which provides a friendly graphical user interface (GUI) to select mathematical models, cardiac rhythms, and parameters. By using a microcontroller STMF103C8T6 operating at 72MHz and belonging to the ARM Cortex-M3 Core family, we can control the pixels on the LCD screen and scan messages from the touch screen through a parallel bus and two resistive terminals, respectively (see Figure 1b). Information scanned from the TFT touch screen by the slave MCU (STMF103C8T6) is sent to the master MCU (STM32F401CCU6) via SPI protocol. These data allow us to specify the mathematical model and cardiac rhythm that will be solved numerically in the master MCU. The output ECG waveforms are converted to a quasi-analog signal by using DACs. Since the DAC operates voltages on the order of millivolts, the internal noise may adversely affect its performance. To aleviate this, we first include a bypass capacitor to minimize the effect of noise sources on signal integrity, and second, we generate ECG waveforms with amplitudes ten times greater than real ECG amplitudes, including offset levels that simulate baselines. In addition, to simulate ECG waveforms with physiologically consistent amplitudes, the output signals of the converters are passed through a non-inverting amplifier stage, encompassing general-purpose operational amplifiers and resistors (R ${}_{1}$, R ${}_{2}$, R ${}_{g}$, and R ${}_{f}$) for producing a differential output signal, as described in Figure 1c. This amplifier provides low offset voltage and low noise. With this design, we can produce ECG waveforms within the range of 0.5 mV to 4 mV with low noise and minimum offset effects.

While the discretized reaction–diffusion model [38], the ring of three coupled oscillators [39], and the model based on a quasiperiodic motion [40] can reproduce only standard Einthoven lead II, the network of heterogeneous oscillators [37] can produce a realistic 12-lead ECG profile. Typically, cardiologists place six electrodes on the patient’s chest and four on the limbs to obtain a 12-lead electrocardiogram, including three bipolar limb leads (Lead *I*, II, and III), three augmented limb leads (aVL, aVF, and aVR), and six precordial leads (V1–V6) ([3], Chapter 12).

The electrodes placed on the right arm (RA), left arm (LA), and left leg (LL) form an equilateral triangle known as Einthoven’s triangle and give place to the bipolar limb leads. The three bipolar limb leads in Einthoven’s triangle, denoted by I, II and III, can be obtained by Equation (11) and satisfy the relationship II=I+III:

(11) $$\begin{array}{cc} I & =LA-RA,\\ II & =LL-RA,\\ III & =LL-LA. \end{array}$$

The augmented limb leads and precordial leads are the electrical potential differences between physical and virtual electrodes [55]. For the augmented limb leads aVF, aVL, and aVR, the physical electrodes correspond to RA, LA, and LL, and the virtual electrode is the so-called Goldberger’s central terminal, whose potential is the mean voltage of two limb electrodes that remain when an electrode is selected. In this way, augmented limb leads can be written as functions of the physical electrodes RA, LA, and LL as follows:

(12) $$\begin{array}{cc} aVR & =RA-\frac{1}{2}\left(LA+LL\right),\\ aVL & =LA-\frac{1}{2}\left(RA+LL\right),\\ aVF & =LL-\frac{1}{2}\left(RA+LA\right). \end{array}$$

Typically, patient monitoring systems, such as electrocardiographs, derive the bipolar and augmented limb leads from the limb electrodes. In this regard, our ECG patient simulator solely generates these three ground-referenced electrical potentials to obtain the six first leads: *I*, II, III, aVL, aVR, and aVF. As shown in Figure 1d, we implement a Wye resistor network to satisfy Equations (11) and (12). The voltages of each terminal in the Wye connection are supplied by three DACs with series MCP4921.

Precordial leads, labeled by V1, V2,…,V6, are unipolar potentials referenced to a common electrode known as the Wilson central terminal. This terminal is a theoretical point computed by the mean voltage of the limb electrodes. By Kirchhoff’s laws, the sum of the electrical potentials in the limb electrodes is zero. So, the Wilson terminal works as the reference point for the six electrodes placed on the chest. In our electronic device, the Wilson center was set to zero, allowing us to directly reproduce the precordial leads by using ground-referenced DACs.

The described ECG patient simulator cannot only generate a full 12lLead profile by numerically integrating the extended heterogeneous oscillator model of the cardiac conduction system but it can also produce a single lead, specifically the standard Einthoven lead II. The user can select the mathematical model in order to specify the arrhythmia through the TFT touch LCD display.

### 2.3. Software

**Figure 2:**
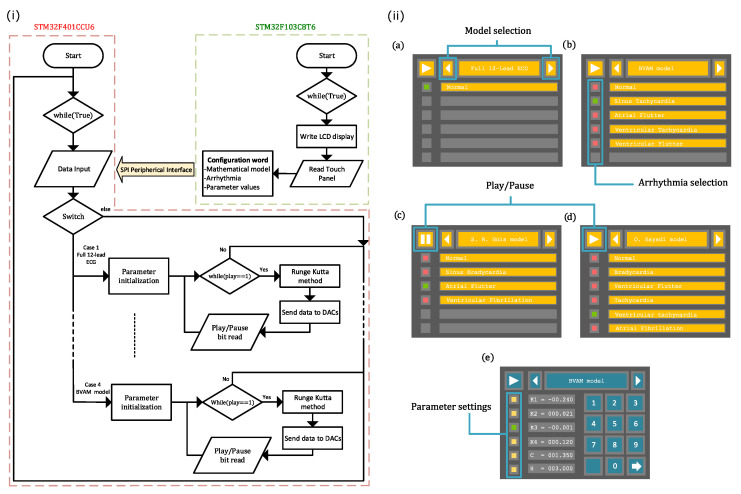
(**i**) Flowchart of the ECG patient simulator for both microcontrollers, STM32F401CCU6 and STM32F103C8T6. (**ii**) Graphical user interface, where different screens to interact with the user were implemented: (**a**) model selection, (**b**) arrhythmia selection, (**c**,**d**) play/pause generation of ECG waveforms, and (**e**) parameter settings.

The algorithms embedded in the STM32F401CCU6 and STM32F103C8T6 microcontrollers are described in what follows. Figure 2i shows the flowchart for both microcontrollers. For an easy configuration of the STM32F401CCU6 and STM32F103C8T6 microcontrollers, we use STM32 Cube MX and STM32 Cube IDE as integrated development environments (IDE). Both of them include a graphical tool that allows setting peripherals, clock, and general-purpose inputs/outputs.

The STM32F103C8T6 manages the user interface (shown in Figure 2ii) in the TFT LCD Screen via an 8-bit parallel protocol and reads the LCD’s resistive touch panel using two analog-to-digital channels. This MCU generates a configuration word from the data introduced by users through the GUI shown in Figure 2ii, whose screens are related to: (a) model selection, (b) arrhythmia selection, and (e) parameter settings. It should be noted that we have preloaded the parameter values for each rhythm so that the user does not have to introduce parameters one by one. This allows them to easily choose a particular ECG waveform through the graphic interface (model selection and arrhythmia selection menus). However, the proposed system can be set by individually changing the parameter values to explore different conditions through the parameter settings menu. The graphical interface also includes some controls to play or pause the generation of ECG waveforms, as displayed in Figure 2ii(c,d). To interface with the LCD display, bits A8–A15 from port A and bits B3–B5 and B12–B15 from port B are configured as push–pull outputs at maximum speed. The STM32F401CCU6 and STM32F103C8T6 microcontrollers communicate with each other via the SPI peripheral. Here, the STM32F401CCU6 microcontroller is a slave transmitter, whose bits A7, A5, and B4 from port A and port B are reserved for MOSI, SCK, and MISO signals, respectively. While Channels IN0 and IN1 from analog-to-digital converter 1 are configured to read $V_{x}$ and $V_{y}$ from the touch interface, bit A6 from port A is set as a push–pull output at maximum speed for the play/pause instruction.

The STM32F401CCU6 microcontroller receives and initializes the user-defined model parameters, which are encoded in the configuration bits provided by the STM32F103C8T6 microcontroller. From this information, STM32F401CCU6 solves the selected mathematical model through the fourth-order Runge–Kutta (RK4) method. We have included the numerical method within a looping function, which checks, in each iteration, the state of the play/pause bit coming from the STM32F103C8T6 microcontroller. The integration step used for solving each model is obtained by measuring the computing time of one iteration plus the time that the MCU takes to send data to the DAC via the SPI peripheral. It is important to say that two of the four models integrated into the ECG patient simulator include time delays in the coupling, which requires solving a set of delayed differential equations. To overcome this issue, we create an array that buffers a certain number of samples from the delayed signal. The amount of samples depends on the ratio of the transport delay terms and the integration step.

Finally, to individually control each MCP4921 DAC’s, Bits A8–A15 from port A and bits B12–B15 from port B are configured as chip select and bit A6 as the latch. The STM32F401CCU6 microcontroller implements an SPI1 peripheral that works as a half-duplex master at 18 MBits/s. To do this, the APB2 Bus is set to 36 MHz, reserving bits A7, A5, and B4 from A and B ports for the MOSI, SCK, and MISO signals, respectively. Bit A8 from port A is configured as an input for the play/pause instruction.

## 3. Results

**Figure 3:**
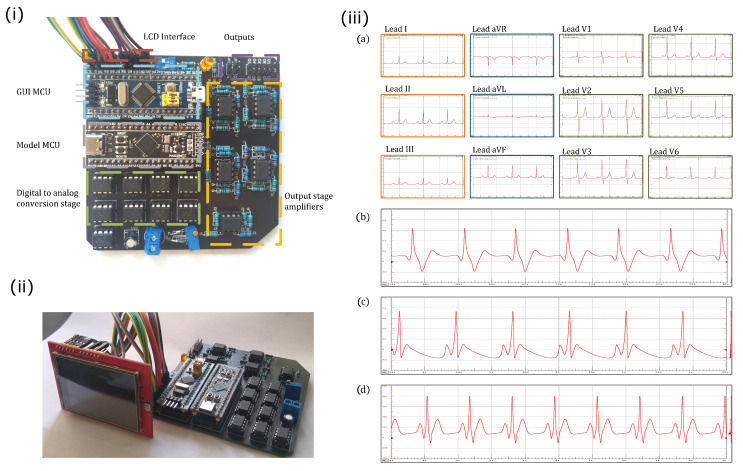
(**i**) Electronic circuit of the proposed ECG patient simulator. (**ii**) Electronic circuit including the TFT LCD touch screen. (**iii**) Normal synthetic ECG waveforms obtained from the ECG patient simulator for different mathematical models: (**a**) network of heterogeneous oscillators (3), (**b**) discretized reaction–diffusion model (5), (**c**) ring of three coupled oscillators (7), and (**d**) model based on a quasiperiodic motion (10).

The microcontroller and passive electronic components were mounted on a two-layered printed circuit board (PCB) to avoid faulty contacts and poor stability. The whole PCB was designed in EasyEDA Software. The ECG patient simulator implemented in the PCB is presented in Figure 3i. The microcontrollers that manage the communication protocol with the TFT LCD touch screen (red square) and the generation of ECG waveforms are indicated with cyan and gray squares, respectively. The green and orange squares indicate the digital-to-analog conversion stage and the output amplifiers, respectively. In the same Figure, the synthetic ECG waveforms are provided through the connector marked with a purple square. Figure 3ii shows the whole electronic circuit, i.e., the core for the generation of ECG signals and the TFT LCD touch screen.

Normal synthetic ECG signals generated by the proposed ECG patient simulator are presented in Figure 3iii. Table 1 shows the parameter values for reproduce these cardiac rhythms. We obtain a full 12-lead ECG profile by integrating the heterogeneous oscillator model of the cardiac conduction system as shown in Figure 3iii(a). An ECG waveform (standard Einthoven lead II) generated with the discretized BVAM model is shown in Figure 3iii(b). The normal ECG waveforms produced by the model based on a ring of three coupled oscillators and the model based on a quasiperiodic motion are shown in Figure 3iii(c,d), respectively. Notably, the ECG signals presented in Figure 3iii(b,d) exhibit the characteristic peaks and troughs of the ECG waveform, i.e., the P wave, QRS complex, and T wave. These events are associated with the successive atrial depolarization/repolarization and ventricular depolarization/repolarization, which occurs with every heartbeat. It should be said that the discretized reaction–diffusion model and the ring of three coupled oscillators can only reproduce general features of the normal ECG, though they are capable of generating different arrhythmias effectively. On the contrary, the network of heterogeneous oscillators and the model based on a quasiperiodic motion incorporate substantial details in the simulated signals that lead to realistic ECG waveforms, but the complexity of the models increases.

**Figure 4:**
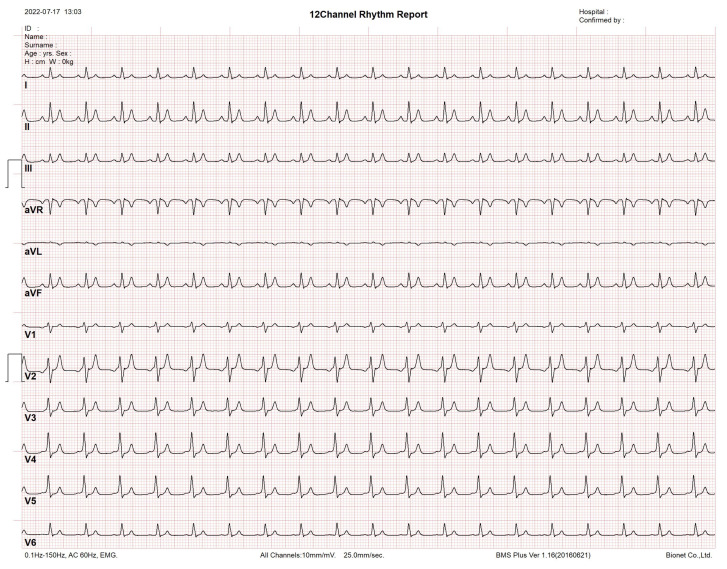
Synthetic 12-lead ECG profile tested using an interpretive 12 channel electrocardiogram machine with series CardioCare 2000.

Since the network of heterogeneous oscillators is the sole model that reproduces a full 12-lead ECG profile, we assess it in a commercial electrocardiograph, which is a monitoring device consisting of an interpretive 12-channel electrocardiogram machine, with series CardioCare 2000. The monitoring equipment recognizes the synthetic ECG signals effectively, as shown in Figure 4.

### 3.1. Rhythm Disorders

In what follows, we show different arrhythmia that the proposed system can reproduce. We would like to emphasize that in most models, the parameter values for the arrhythmias are the same as for the normal state. So, in the subsequent sections, we will only specify those parameters that change.

#### 3.1.1. Network of Heterogeneous Oscillators

**Figure 5:**
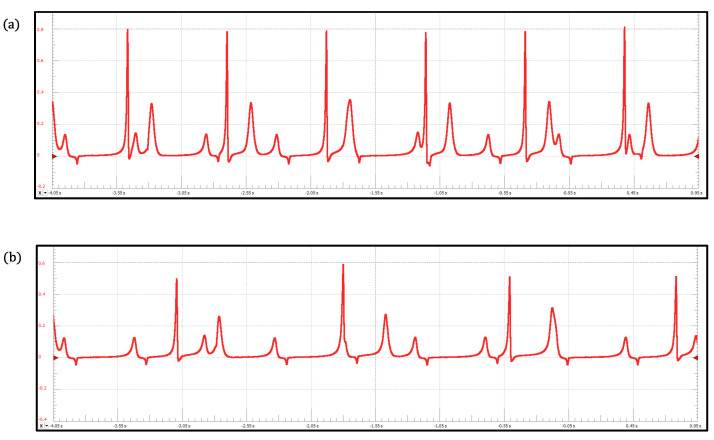
ECG waveforms obtained with the heterogeneous oscillator model of the cardiac conduction system: (**a**) Complete SA–AV block and (**b**) Complete AV–HP block.

To validate the proposed ECG simulator, we first reproduce two well-known rhythm disorders, complete SA–AV and AV–HP blocks, by integrating the heterogeneous oscillator model of the cardiac conduction system (3). The results are shown in Figure 5. A complete SA–AV block indicates the loss of communication between the SA node and the AV node. Here, the SA node cannot control the heart rate, leading to a lack of coordination of the depolarization/repolarization processes in the atria and ventricles. We reproduced the complete SA–AV block by setting the coupling constant $K_{SA-AV}=0$. In contrast, for the complete AV–HP block ($K_{AV-HP}=0$), the HP complex acts independently at its own rate and the SA and AV nodes operate coupled at a normal rhythm. Consequently, atria work normally, and ventricles take action at a slow rate.

#### 3.1.2. Reaction–Diffusion Model Spatially Discretized

**Figure 6:**
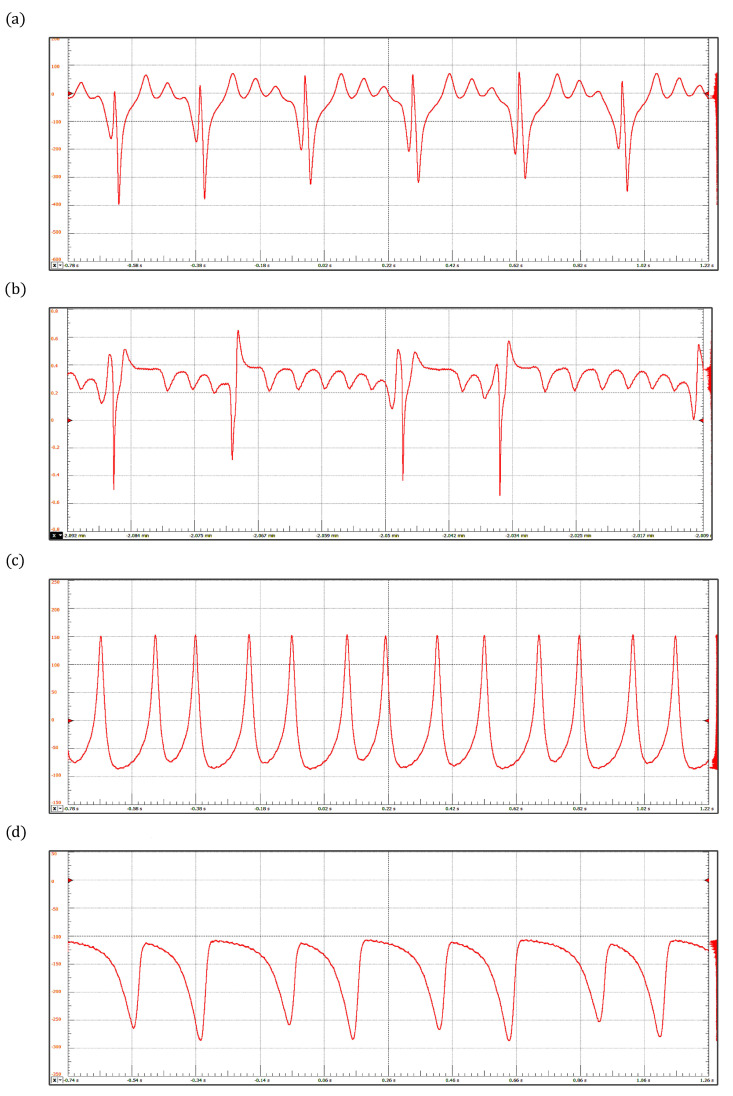
ECG waveforms obtained with the reaction-diffusion model: (**a**) Sinus Tachycardia, (**b**) Atrial Flutter, (**c**) Ventricular Tachycardia, and (**d**) Ventricular Flutter.

Figure 6 shows four arrhythmias generated by the discretized reaction–diffusion model: (a) sinus tachycardia, (b) atrial flutter, (c) ventricular tachycardia, and (d) ventricular flutter. The corresponding parameter values are presented in Table 2. Sinus tachycardia is a cardiac rhythm characterized by a faster-than-usual heartbeat. Similarly, atrial flutter refers to a condition in which the upper chambers beat too quickly. However, the former condition is generally not dangerous, but the second one produces insufficient blood pumping, which can lead to heart failure [56,57]. Lethal cardiac arrhythmias frequently result from reentry mechanisms; specifically, ventricular tachycardia and ventricular flutter are re-entrant ventricular tachyarrhythmias that can progress to ventricular fibrillation. Ventricular tachycardia occurs when the lower chamber of the heart (ventricles) beats faster than 120 beats/min, whereas ventricular flutter is a rapid organized rhythm, between 250 and 300 beats/min, characterized by a sine wave pattern on the electrocardiogram without any identifiable QRS complexes or T waves [58,59].

#### 3.1.3. Ring of Three-Coupled Oscillators

**Figure 7:**
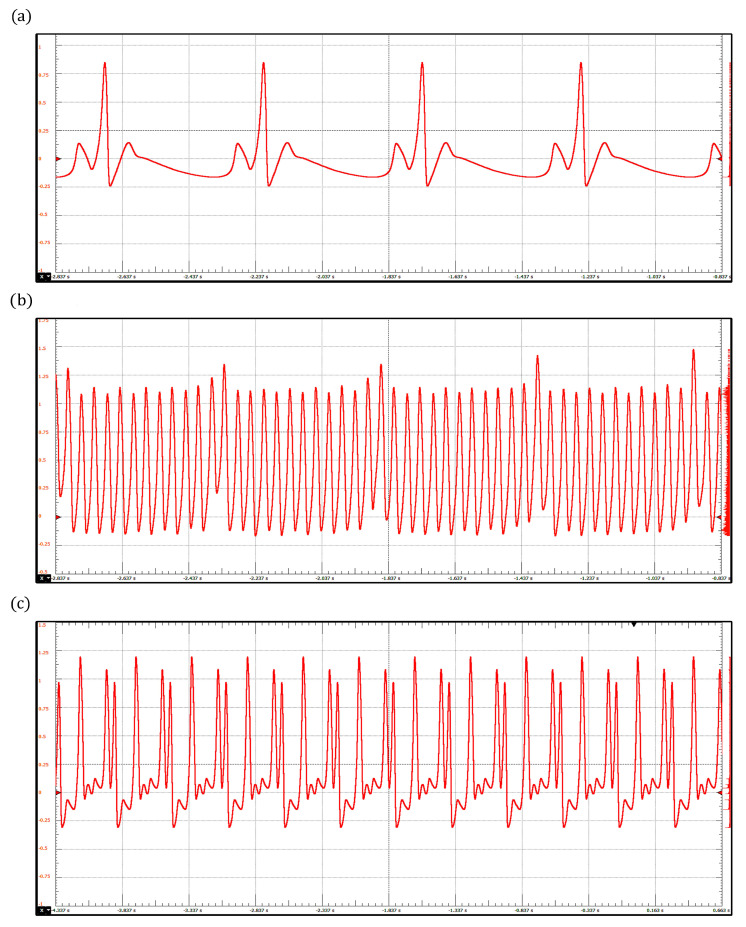
ECG waveforms obtained with the ring of three-coupled oscillators model: (**a**) Sinus Bradycardia, (**b**) Atrial Flutter, and (**c**) Ventricular Fibrillation.

By integrating the model (6) in the proposed device, we reproduce three pathological rhythms: sinus bradycardia, atrial flutter, and ventricular fibrillation, which are shown in Figure 7. Sinus bradycardia is a common heart rhythm defined by a slow, regular heartbeat of fewer than 60 beats/min [56]. In contrast, ventricular fibrillation is a severe and totally disorganized rhythm that leads to death if no immediate medical attention is provided [59]. Interestingly, ventricular fibrillation has been associated with chaotic behavior; this fact has motivated the investigation and development of new devices to control it. In this context, ECG simulators have provided a significant tool to design and test these devices [60,61,62]. The parameter values corresponding to the three pathologies are shown in Table 3.

#### 3.1.4. Extended Dynamical Model Based on a Quasi-Periodic Motion

**Figure 8:**
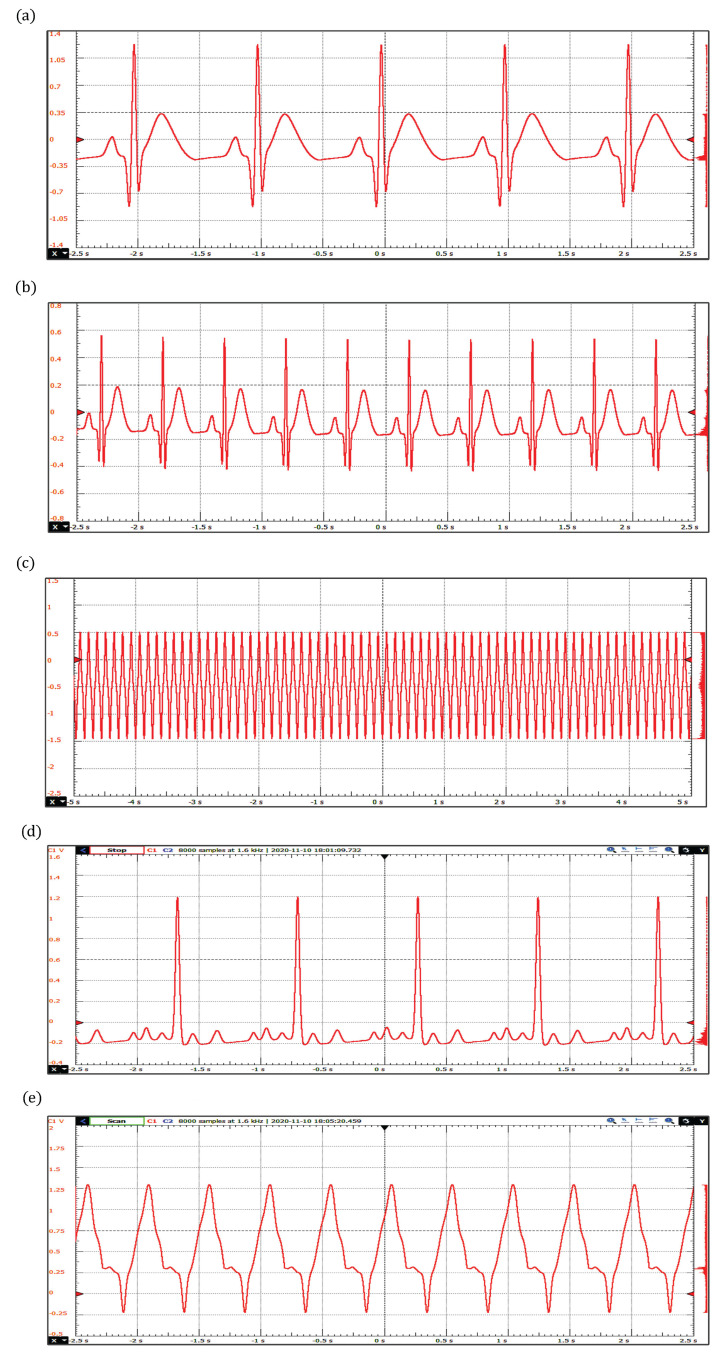
ECG waveforms obtained with the extended dynamical model based on a quasi-periodic motion: (**a**) Bradycardia, (**b**) Tachycardia, (**c**) Ventricular Flutter, (**d**) Atrial Fibrillation, and (**e**) Ventricular Tachycardia.

In Figure 8, arrhythmias generated with the extended dynamical model based on a quasi-periodic motion are shown. These include sinus bradycardia, sinus tachycardia, ventricular flutter, atrial fibrillation, and ventricular tachycardia. The corresponding parameter values are presented in Table 4. As mentioned above, sinus bradycardia and sinus tachycardia are not serious arrhythmias, in fact, they often do not require treatment. However, there are abnormalities of cardiac rhythm that are potentially dangerous. For example, atrial fibrillation causes irregular heartbeats that begin in the upper chambers and can produce blood clots, limiting the ability to pump blood into the body.

We would like to stress that the proposed ECG simulator can generate a wide range of pathological conditions, so it provides a promising platform not only to train arrhythmia classifiers and detectors [63,64] but also to assess ECG signal processing software. Signal denoising is a remarkable example where synthetic waveforms can help attenuate the noise of real ECG signals by providing a reference profile to wave detector [65].

## 4. Discussion

In the last years, synthetic ECG patient simulators have been developed to test, calibrate, and assess electrocardiograph equipment and ECG waveform processing software. Most of these simulators have shortcomings in the amplitudes, range, waveforms, and time-intervals of the produced signals. To overcome these shortcomings, and in light of the importance of mathematical modeling for the understanding of complex biological processes involved in the cardiac conduction system, we introduced a versatile, low-cost, and tunable electronic device implementing an ECG patient simulator based on four mathematical models to generate realistic ECG waveforms within a broad range of possibilities. It is worth mentioning that the ECG profiles generated by the models incorporated in our ECG patient simulator were already compared with clinical registers in the corresponding works where they were published. In particular, the network of heterogeneous oscillators and the model based on a quasiperiodic motion incorporate substantial details in the simulated ECG signals that lead to realistic waveforms. Furthermore, the discretized reaction–diffusion model and the ring of three coupled oscillators can generate different arrhythmias effectively by changing a few parameters. One of the most significant features of our ECG patient simulator is the possibility of controlling parameters of the mathematical models through an interactive and friendly graphic interface. In addition, the proposed ECG patient simulator is able to produce a full 12-lead ECG profile, which can be tested in patient monitoring systems. We are certain that our proposal constitutes a promising platform for testing medical equipment and biological signal processing algorithms, as well as a powerful tool for medical education and academic research. The proposed system can be considered as an alternative to calibrate, test, and certify ECG devices in order to confirm the level of confidence in the manufacturers’ specifications. Future research directions could be the training and evaluation of automated diagnosis systems and the development of a cardiac defibrillator to control, in an efficient way, irregular and chaotic heartbeats associated with ventricular fibrillation.

## Figures and Tables

**Table 1:** Parameter values for reproducing normal rhythms using different models. In the model based on a quasiperiodic motion, the parameters of the Gaussian kernels are expressed by the values of $\left(a_{i},b_{i},\theta_{i}\right)$ for each characteristic waveform.

Pathology	Parameters
Network of heterogeneous oscillators	$a_{1}=40$, $a_{2}=a_{3}=50$, u=0.69, $K_{SA-AV}=K_{AV-HP}=f_{1}=22$, $f_{2}=8.4$, $f_{3}=1.5$, d=3, $e_{1}=3.5$, $e_{2}=5$, $e_{3}=12$, $\tau_{SA-AV}=\tau_{AV-HP}=0.092$, $k_{1}=2\times 10^{3}$, $k_{2}=1\times 10^{2}$, $k_{3}=1\times 10^{4}$, $k_{4}=2\times 10^{3}$, $c_{1}=0.26$, $c_{2}=c_{3}=0.12$, $c_{4}=0.1$, $b_{1}=b_{2}=b_{4}=0$, $b_{3}=0.015$, $g_{1}=0.4$, $g_{2}=g_{3}=0.09$, $g_{4}=0.1$, $h_{1}=0.004$, $h_{2}=h_{3}=h_{4}=0.008$, $w_{11}=0.13$, $w_{12}=1.0$, $w_{21}=w_{31}=0.12$, $w_{22}=w_{32}=1.1$, $w_{41}=0.22$, $w_{42}=0.8$, $C_{1}=4\times 10^{-5}$, $C_{2}=-4\times 10^{-5}$, $C_{3}=9\times 10^{-5}$, $C_{4}=-6\times 10^{-5}$ and $z_{0}=0.2$
Discretized reaction–diffusion model	C=1.35, β=4, H=3, $K_{1}=-0.024$, $K_{2}=0.0216$, $K_{3}=-0.0012$, $K_{4}=0.12$ and $\Gamma_{t}=7$.
Ring of three coupled oscillators	$\alpha_{0}=1$, $\alpha_{1}=0.1$, $\alpha_{3}=0.05$, $\alpha_{5}=0.4$, $\alpha_{SA}=3$, $W_{SA_{1}}=0.2$, $W_{SA_{2}}=-1.9$, $d_{SA}=3$, $\alpha_{AV}=3$, $W_{AV_{1}}=0.1$, $W_{AV_{2}}=-0.1$, $d_{AV}=3$, $e_{AV}=3$, $\tau_{SA-AV}=\tau_{AV-HP}=\tau_{SA-HP}=0$, $\tau_{HP-SA}=0$, $\tau_{AV-SA}z=0.8$, $\tau_{HP-SA}=0$, $k_{SA-AV}=k_{AV-HP}=k_{SA-HP}=0,k_{HP-SA}=0$, $\rho_{SA}=1$, $\rho_{AV}=1$, $\rho_{HP}=20$, $k_{HP-SA}=0$, $e_{SA}=4.5$, $k_{AV-SA}=5$, $k_{HP-AV}=20$, $W_{SA}W_{AV}=W_{HP}=0$, $\beta_{T}=16$ and $\beta_{G}=0.0012$.
Model based on a quasiperiodic motion	$P^{+}\left(1.2,0.25,-\pi /3\right)$, $P^{-}\left(0,0.25,-\pi /3\right)$, Q(−0.5,0.1,−π/12), R(30,0.1,0), S(−7.5,0.1,π/12), $T^{+}\left(0.75,0.45,\pi /2\right)$ and $T^{-}\left(0,0.75,\pi /2\right)$.

**Table 2:** Parameter values for the reaction–diffusion model.

Pathology	Parameters
Sinus Tachycardia	H=2.848, $K_{1}=0$, $K_{2}=-0.1$, $K_{3}=0$, $K_{4}=0$, $\Gamma_{t}=21$
Atrial Flutter	H=1.52, $K_{1}=0.068$, $K_{2}=0.028$, $K_{3}=0.024$, $K_{4}=0.012$, $\Gamma_{t}=13$
Ventricular Tachycardia	H=2.178, $K_{1}=0$, $K_{2}=0$, $K_{3}=0$, $K_{4}=-0.1$, $\Gamma_{t}=21$
Ventricular Flutter	H=2.178, $K_{1}=0.1$, $K_{2}=-0.02$, $K_{3}=-0.01$, $K_{4}=0$, $\Gamma_{t}=13$

**Table 3:** Parameter values for the ring of three-coupled oscillators.

Pathology	Parameters
Ventricular Flutter	$e_{SA}=4.5$, $k_{AV-SA}=0$, $k_{HP-AV}=20$, $W_{SA}=0$, $W_{AV}=0$, $W_{HP}=0$, $\beta_{T}=8$ and $\beta_{G}=0.0012$
Sinus Bradycardia	$e_{SA}=4.5$, $k_{AV-SA}=5$, $k_{HP-AV}=15$, $W_{SA}=0$, $W_{AV}=0$, $W_{HP}=0$, $\beta_{T}=8$ and $\beta_{G}=0.0009$
Ventricular Fibrillation	$e_{SA}=6$, $k_{AV-SA}=5$, $k_{HP-AV}=20$, $W_{SA}=7.33$, $W_{AV}=7.33$, $W_{HP}=7.33$, $\beta_{T}=16$ and $\beta_{G}=0.0012$

**Table 4:** Parameter values for the extended dynamical model based on a quasiperiodic motion. The parameters of the Gaussian kernels are expressed by the values of $\left(a_{i},b_{i},\theta_{i}\right)$ for each characteristic waveform.

Waves	Sinus Bradycardia	Sinus Tachycardia	Ventricular Flutter	Atrial Fibrillation	Ventricular Tachycardia
$P^{-}$	(0.7,0.2,−3π/8)	(0.7,0.2,−3π/7)	(0,0.1,−π/6)	(0.7,0.12,−5π/7)	(1,0.2,10π/13)
$P^{+}$	(0.8,0.1,−π/3)	(0.8,0.1,−π/3)	(0,0.1,−2π/3)	(0.9,0.13,−π/2)	(1,0.1,−2π/3)
*Q*	(−1,0.1,−π/13)	(−7,0.1,−π/13)	(0,0.1,−π/12)	(0.6,0.12,−π/4)	(−12,0.2,−π/3)
*R*	(20,0.1,0)	(20,0.1,0)	(20,0.6,−π/2)	(18,0.1,0)	(1,0.3,0)
*S*	(−9.5,0.1,π/15)	(−9.5,0.1,π/17)	(−20,0.6,π/2)	(−0.1,0.05,−π/30)	(3,0.4,2π/11)
$T^{-}$	(0.27,0.4,2π/5)	(0.27,0.4,π/2)	(0,0.1,3π/8)	(0.62,0.15,π/4)	(5,0.5,π/2)
$T^{+}$	(0.15,0.55,4π/7)	(0.15,0.55,4π/7)	(0,0.1,5π/8)	(0.55,0.17,7π/11)	(3,0.45,2π/23)

## Data Availability

Data underlying the results presented in this paper are not publicly available at this time but may be obtained from the authors upon reasonable request.

## Abbreviations

The following abbreviations are used in this manuscript:

ECG	Electrocardiogram
RK4	Fourth-order Runge-Kutta
DAC	Digital-to-analog converter
OPAMP	Operational amplifier
SA	Sinoatrial
AV	Atrioventricular
HP	His–Purkinje
BVAM	Barrio–Varea–Aragon–Maini
VdP	Van der Pol
VdM	Van der Mark
MCU	Microcontrollers
GUI	Graphical user interface
USB	Universal serial bus
SPI	Serial peripheral interface
I2C	Inter-integrated circuit
USART	Universal synchronous and asynchronous serial receiver and transmitter
CAN	Controller area network
TFT LCD	Thin-film-transistor liquid-crystal display
DC	Direct current
SCK	Serial clock
MOSI	Master out slave in
MISO	Master in slave out
PCB	Printed circuit board
